# Inflammatory CD4^+^ T cells can waive NRF2-dependent SLC7A11-mediated cystine uptake by using ASCT1

**DOI:** 10.1016/j.isci.2026.115680

**Published:** 2026-04-12

**Authors:** Christopher Thomas Neullens, Sudheendra Hebbar Subramanyam, Gerd Horneff, Tilmann Kallinich, Freya Huijsmans, Jorg van Loosdregt, Bas Vastert, Klaus Tenbrock, Kim Ohl

**Affiliations:** 1 Department of Pediatrics, Pediatric Rheumatology, Medical Faculty, RWTH Aachen University, Aachen, Germany; 2 Sankt Augustin Children`s Hospital, Pediatric Rheumatology, Sankt Augustin, Germany; 3 Department of Paediatric Respiratory Medicine, Immunology and Critical Care Medicine, Charité - Universitätsmedizin Berlin, Deutsches Rheuma-Forschungszentrum (DRFZ), Institute of the Leibniz Association, Berlin and German Center for Child and Adolescent Health, Berlin Site, Berlin, Germany; 4 UMC Utrecht, Pediatric Rheumatology and Immunology, Utrecht, the Netherlands; 5 Department of Paediatrics, Pediatric Rheumatology, Inselspital, Bern University Hospital, University of Bern, Bern, Switzerland; 6 Institute of Immunology, Medical Faculty, RWTH Aachen University, Aachen, Germany; 7 Faculty of Food and Nutrition Sciences, University of Applied Sciences, Hochschule Niederrhein, Moenchengladbach, Germany

**Keywords:** Biochemistry, Immunology, Cell biology

## Abstract

Ferroptosis and lipid peroxidation are associated with inflammatory and pathogenic conditions. However, cell-specific mechanisms and functions are not fully understood. Cysteine is an essential amino acid for T cell activation and proliferation and is required to synthesize glutathione, the most abundant antioxidant molecule in cells. Cysteine is predominantly produced intracellularly after uptake of its oxidized form (cystine) by SLC7A11. In this study, we provide a detailed analysis of lipid peroxidation in human T cells and analyzed functional consequences in the chronic inflammatory condition of childhood arthritis. We found that healthy peripheral blood CD4 T cells are not fully dependent on SLC7A11 expression and cystine uptake to prevent ferroptotic cell death, most likely by switching to ASCT1-mediated cysteine uptake. T cells from patients with JIA have a high ASCT1 expression, which most likely prevents exaggerated lipid peroxidation and enables them to maintain their inflammatory phenotype in challenging environments such as inflamed joints.

## Highlights

• T cells can upregulate ASCT1 to bypass SLC7A11-dependent cystine uptake
• Enhanced lipid peroxidation coexists with high inflammatory cytokine production
• Switching to ASCT1/cysteine uptake might preserve functions in challenging environments

## Graphical abstract

**Figure:**
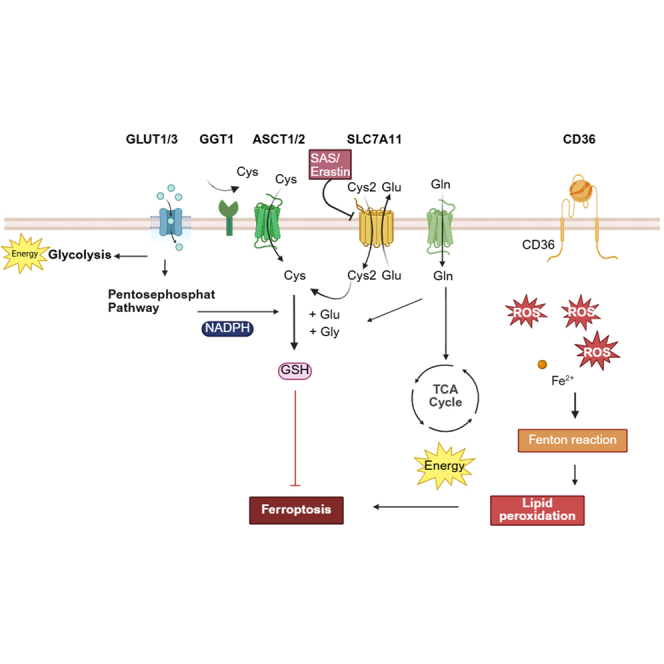

## Introduction

Ferroptosis is a type of regulated cell death that is dependent on reactive oxygen species (ROS) and is associated with lipid peroxidation and iron accumulation. Ferroptosis occurs through two main pathways. The extrinsic/transporter-dependent pathway (mediated by solute carrier family 7 member 11 (SLC7A11)) and the intrinsic/enzyme-regulated pathway (mediated by glutathione peroxidase 4 (GPX4) and arachidonate lipoxygenases (ALOXs)). CD36, a key receptor involved in fatty acid (FA) uptake, can also contribute to lipid peroxidation and ferroptosis.^1^ In addition, there are several other factors that influence ferroptosis, allowing us to conceptualize it as a regulated cell death process that is tightly controlled at multiple levels.^2^

Cystine (Cys_2_) uptake is mediated by cystine transporter SLC7A11 (also called xCT). Once imported into the cytosol, cystine is reduced to cysteine (Cys), which is subsequently used to synthesize glutathione (GSH) for antioxidant defense. Most of the pathways involved in ferroptosis are under the transcriptional regulation of nuclear factor erythroid 2-like 2 (NRF2), including GSH regulation, iron regulation (such as ferritin heavy chain 1 (FTH1), ferritin light chain (FTL) and ferrous iron exporter ferroportin 1 (FPN1)), NADPH regeneration (such as glucose-6-phosphate dehydrogenase (G6PD) and ME1 (malic enzyme 1)), SLC7A11 and GPX4 and so on.^3^ Inhibition of SLC7A11 with the small molecule compound erastin or the drug sulfasalazine (SAS) intervenes these pathways and induces ferroptosis.^4^^,^^5^

Ferroptosis has been implicated in a variety of pathological contexts, including cancer, (neuro)degenerative diseases, but also in immune-mediated diseases such as non-alcoholic steatohepatitis (NASH), diabetes, and asthma.^6^ The pro-inflammatory effects are due to the release of damage-associated molecular patterns (DAMPs) (e.g., high-mobility group box 1 (HMGB1)) in the environment. However, cell-specific functions of ferroptosis, especially for T cells, remain less clear.^7^ So far, it is only known that redox metabolism is critical for T cell functions. Low levels of ROS are associated with activation, proliferation, and effector functions, while elevated ROS must be neutralized by antioxidants such as GSH to allow T cells to survive and proliferate.^8^ Cys, the rate-limiting substrate for GSH synthesis, is an essential amino acid for T cells, as they are not equipped for its synthesis and therefore require the cystine uptake by cystine transporters such as SLC7A11.^9^

CD4^+^ T cells play a dominant role in the inflammatory response in the joints of patients with childhood arthritis (juvenile idiopathic arthritis/JIA).^10^^,^^11^ We have recently shown higher mitochondrial ROS and higher total ROS in synovial fluid (SF) T cells of these patients, as well as reduced NRF2 expression.^12^^,^^13^

We hypothesized that T cells in inflamed tissues have significantly increased requirements for antioxidant defense and therefore are much more dependent on SLC7A11-mediated cystine uptake to obtain Cys and to maintain redox homeostasis than in normal tissues, analogous to oncogene addiction in cancer development. In contrast, we found that T cells from the inflamed joints of patients with JIA have reduced SLC7A11 expression. Also, healthy peripheral T cells upon SLC7A11 inhibition or downregulation do not undergo excessive ferroptotic cell death, most likely by switching to neutral amino acid exchanger 1 (ASCT1)-mediated Cys uptake. T cells from JIA patients have a high ASCT1 expression, which most likely prevents exaggerated ferroptosis and enables them to maintain their inflammatory phenotype.

## Results

### Inhibition of the NRF2/SLC7A11-dependent ferroptosis pathway downregulates cystine uptake; however, it does not induce cell death

**Figure 1:**
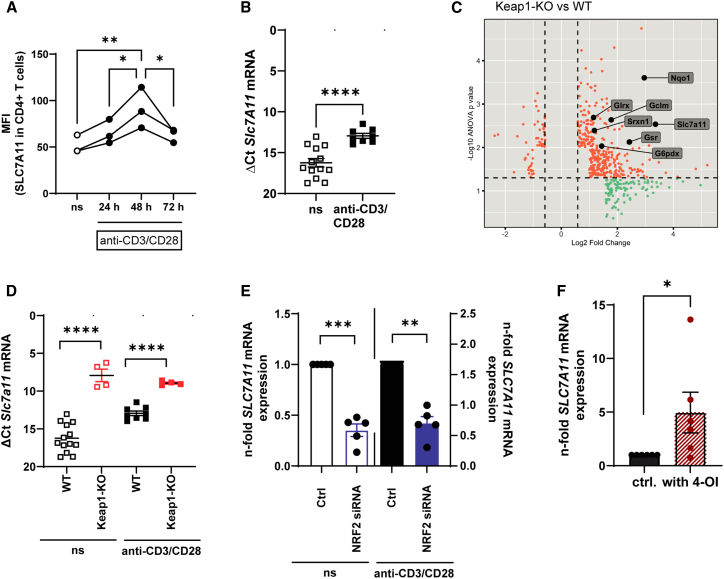
Regulation of lipid peroxidation is extrinsically regulated by the SLC7A11 pathway in CD4^+^ T cells (A) Statistical analysis of SLC7A11 protein expression assessed by flow cytometry (shown as mean fluorescent intensity (MFI). CD4^+^ T cells were either left unstimulated (control) or measured at a distinct time point after stimulation with anti-CD3/CD28 antibodies. *N* = 3, 3 independently performed experiments, a one-way ANOVA multiple comparison test was performed. The calculated power of this experiment is 0.84. (B) ΔCT of *SLC7A11* mRNA expression in human CD4^+^ T cells with and without anti-CD3/CD28 stimulation. *N* = 13, 4 independently performed experiments. (C) Gene expression in CD4^+^ T cells from WT and *VAV*^*cre*^*Keap*^*fl/fl*^ (Keap1-KO) mice was assessed by microarray analysis. Colors indicate significant upregulation (red), or downregulation (green) compared to WT. (D) ΔCT of *SLC7A11* mRNA expression in CD4^+^ T cells of WT and Keap1-KO mice, either left unstimulated or stimulated with anti-CD3/CD28 antibodies, 4 independently performed experiments. The calculated power of this experiment is 0.99 for the stimulated and unstimulated groups. (E) N-fold *SLC7A11* expression in human CD4 T cells, either transfected with a control siRNA (Ctrl) or NRF2 siRNA. 3 independently performed experiments were performed. (F) N-fold *SLC7A11* mRNA expression in human CD4 T cells treated with 4-OI or vehicle (Ctrl) *N* = 6, 4 independently performed experiments. A paired Student's t-test was performed comparing the groups of B–F. Data are presented as mean, error bars present ± SEM for all the presented graphs in this figure. Values were considered significant if ∗*p* < 0.05, ∗∗*p* < 0.01, ∗∗∗*p* < 0.001, and ∗∗∗∗*p* < 0.0001. N represents the number of biological replicates.

First, we investigated the SLC7A11/ferroptosis pathway in activated CD4^+^ T cells. The overall gating strategy and examples for ferroptosis measurements can be found in Figure S1. As shown previously, SLC7A11 is upregulated in CD4^+^ T cells after activation in humans (Figure 1A) and mice (Figure 1B).^14^ *SLC7A11* expression is transcriptionally regulated by NRF2. Mice carrying a constitutive activation of NRF2 in immune cells (VAV^cre^Keap^fl/fl^, here depicted as Keap1-KO) showed an exceedingly high *SLC7A11* expression compared to WT mice in a microarray analysis (Figure 1C) and higher RNA expression in unstimulated and anti-CD3/CD28 stimulated CD4^+^ T cells (Figure 1D). We further confirmed that NRF2 regulates *SLC7A11* expression in human T cells,^14^ as siRNA transfection of human peripheral blood mononuclear cells (PBMCs) downregulated *SLC7A11* expression (Figure 1E), whereas treatment with the NRF2 inducer 4-octly-itaconate (4-OI) upregulated *SLC7A11* expression (Figure 1F).^12^

**Figure 2:**
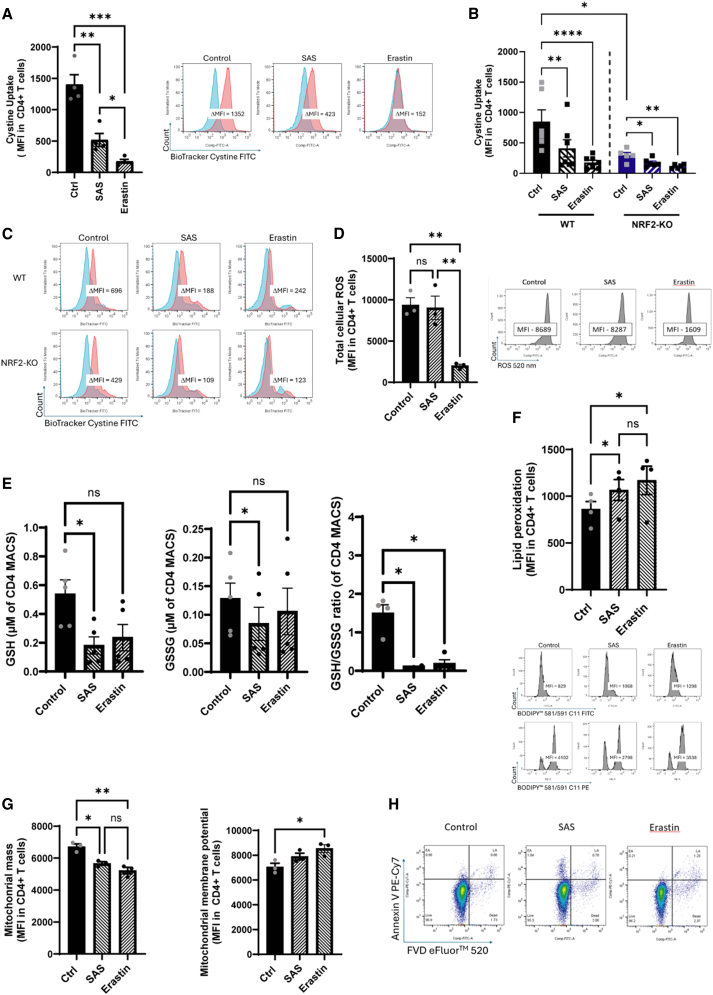
SAS and erastin treatment reduce cystine uptake but not GSH content and cell viability in anti-CD3/CD28 stimulated CD4^+^ T cells (A) Flow cytometric analysis of cystine uptake measured by BioTracker Cystine-FITC in stimulated CD4^+^ T cells in the presence of SAS and erastin. Exemplary histogram, showing the cystine uptake after 0 min (blue) and after 30 min (red) of incubation, while the difference presented as ΔMFI was used to describe the cystine uptake. *N* = 4, 3 independently performed experiments. The calculated power of this experiment is 0.99. (B) Statistical analysis of cystine uptake (represented as MFI) of mouse WT and NRF2-KO CD4^+^ T cells with and without the addition of SAS and erastin. *N* = 6, 4 independently performed experiments. (C) Representative histograms of B showing the uptake after 0 min (blue) and 30 min (red), while the difference presented as ΔMFI was used to describe the cystine uptake. (D) Flow cytometric analysis of reactive oxygen species (ROS) (presented as MFI) of CD4^+^ T cells without and with the addition of SAS and erastin. Histograms of the measurements are shown on the right. The calculated power of this experiment is 0.986. (E) Flow cytometric analysis of intracellular GSH content (presented as ΔMFI) of CD4^+^ T cells with and without SAS and erastin. Histograms depict an overlay of the FMO (blue) and the GSH staining (red). GSH was determined by the GSH/GSSG Luminescence measurement of CD4 MACS HC PBMCs. The kit provides direct analysis of the supernatants' GSH and GSSG contents, which enables the calculation of the ratios. (F) Lipoperoxidation measurement using BODIPY 581/591 C11 (presented as MFI of the FITC signal) of CD4^+^ T cells without and with inhibition by SAS and erastin. Histograms of the two signals, i.e., PE and FITC, from BODIPY are shown on the right. The calculated power of this experiment is 0.94. (G) Flow cytometric analysis of Mitotracker (represented as MFI, mitochondrial mass) of CD4^+^ T cells and TMRM (represented by MFI, mitochondrial membrane potential) of Mitotracker positive cells is shown without and with the addition of SAS and erastin. The calculated power of this experiment is 0.99 for the mitochondrial mass and 1 for the mitochondrial membrane potential. (H) Fluorescent live/dead staining was evaluated by flow cytometry using the combinatory staining of Annexin V and fixable viability dye (FVD) without and with the addition of SAS and erastin. *N* = 3, 3 independent experiments were performed for the graphs (D) to (G). The statistical evaluation of all the represented graphs was performed using one-way ANOVA multiple comparison. (Data are presented as mean, error bars present ± SEM for all the presented graphs in this figure. Values were considered significant if ∗*p* < 0.05, ∗∗*p* < 0.01, ∗∗∗*p* < 0.001, and ∗∗∗∗*p* < 0.0001. N represents the number of biological replicates.

To test whether SLC7A11 is important to prevent ferroptosis in T cells, we used erastin and SAS, both of which are potent and selective inhibitors of SLC7A11. Examples for different gating strategies of the metabolic dyes can be found in Figure S2. Indeed, we found a reduced cystine uptake in human and murine anti-CD3/CD28 stimulated CD4^+^ T cells after SAS and erastin treatment (Figures 2A–2C). Interestingly, T cells of Nrf2-KO mice naturally exhibited a reduced cystine uptake. This was further reduced by incubation with both substances (Figures 2A–2C). Surprisingly, we measured no or reduced levels of cellular ROS (Figure 2D), although SAS and erastin reduced the availability of GSH and turned the GSH/GSSG ratio toward GSSG, suggesting reduced antioxidative capacity (Figure 2E). Lipid peroxidation could be induced by both components (Figure 2F); however, mitochondrial membrane potential, as measured by TMRM in Mitotracker positive cells, showed an increase, while the total mitochondrial mass was reduced (Figure 2G). Live/dead staining showed no evidence of increased cell death (Figure 2H). This suggests that SLC7A11 inhibition induces lipid peroxidation but does not automatically induce ferroptosis-mediated cell death in T cells.

### SLC7A11 inhibition dampens proinflammatory T cells

**Figure 3:**
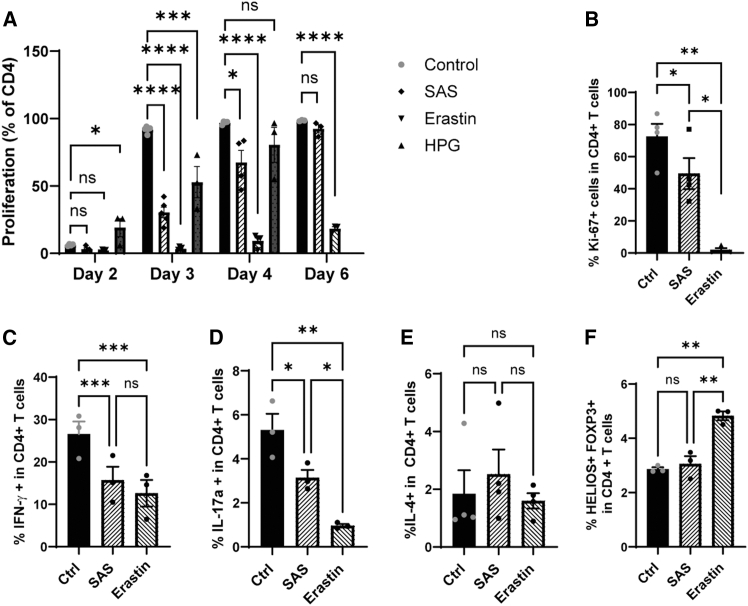
Inhibition of SLC7A11 by SAS and erastin reduces the proliferation of T cells and the release of inflammatory cytokines (A) CD4^+^ T cells were labeled with cell proliferation dye and left unstimulated or stimulated with anti-CD3/CD28 for the indicated time points. Percentages of proliferated cells were determined by flow cytometry. Statistical analysis of *N* = 3–5, 5 independently performed experiments. The calculated power is 0.998 for day 2 and 1 for the rest of the measurements. (B) CD4^+^ T cells were stimulated with anti-CD3/CD28 without (control) or with SAS and erastin, and percentages of Ki-67+ cells were determined by flow cytometry. Statistical analysis of Ki-67+ cells with *N* = 4, 4 independently performed experiments. The calculated power of this experiment is 0.99. (C–F) CD4^+^ T cells were stimulated with anti-CD3/CD28 without (control) or with SAS or erastin, and percentages of IFN-γ+ (C), IL17+ (D), IL4+ (E), and HELIOS+Foxp3+ (F) T cells were determined by flow cytometry. Statistical analysis was performed with *N* = 3, 3 independently performed experiments, and *N* = 4, 4 independently performed experiments (E). For A-F, statistical analysis was performed using One-way ANOVA multiple comparison. The calculated power of the experiment is 0.83 (C), 0.99 (D), 0.11 (E), 0.99 (F). Data are presented as mean, error bars present ± SEM. Values were considered significant if ∗*p* < 0.05, ∗∗*p* < 0.01, ∗∗∗*p* < 0.001, and ∗∗∗∗*p* < 0.0001. N represents the number of biological replicates.

We next asked whether treatment with SAS or erastin alters CD4^+^ T cell function. CD4^+^ T cell proliferation was significantly decreased in the presence of erastin and slowed in the presence of SAS along with the ASCT1 inhibitor 4-Hydroxy-L-phenylglycin (further called HPG) (Figure 3A). Influences on proliferation from SFMCs can be found in Figure S3B. In line with this, erastin completely and SAS slightly downregulated Ki-67 (Figure 3B). Furthermore, SAS and erastin downregulated IFN-γ and IL-17α production of anti-CD3/CD28-stimulated CD4^+^ T cells. Interestingly IL-4 production was not affected, and Helios-Foxp3+ T cells were even enhanced in the presence of erastin (Figures 3C–3F).

We conclude that SAS/erastin-mediated SLC7A11 inhibition was associated with lower proliferation and cytokine production by T cells.

### JIA T cells reveal an aberrant lipid peroxidation pathway

**Figure 4:**
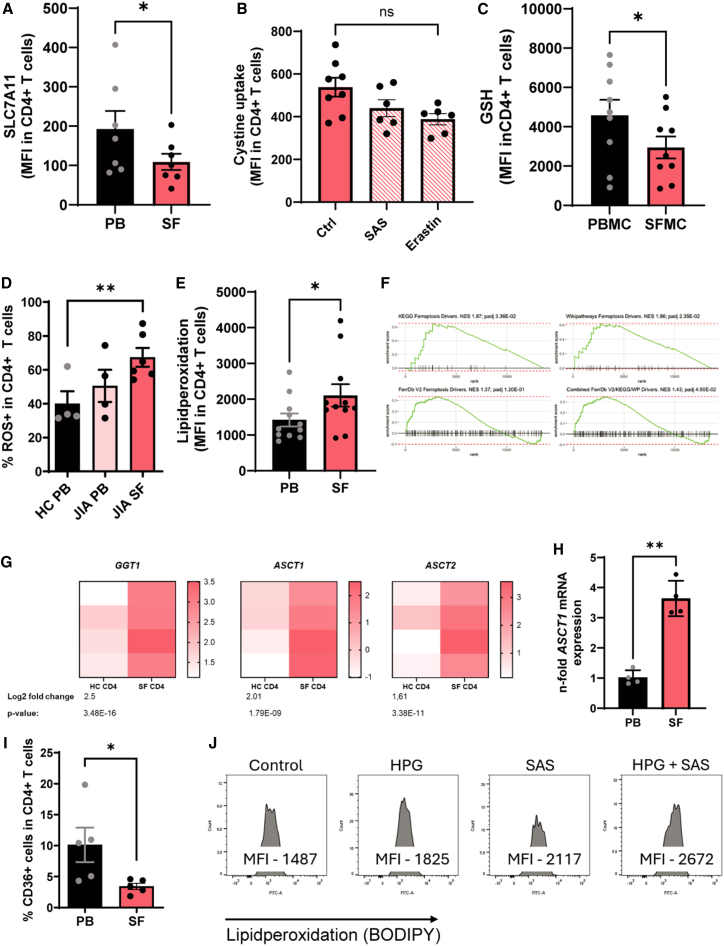
Characterization of an altered NRF2/cystine pathway in patients with JIA (A) Flow cytometric analysis of SLC7A11 expression in stimulated SF and PB CD4^+^ T cells. Statistical analysis was performed with *N* = 7 donors, 7 independently performed experiments. (B) Flow cytometric evaluation of cystine uptake (shown as MFI) by anti-CD3/CD28 stimulated CD4^+^ SF T cells. *N* = 6, 6 independently performed experiments. (C) Flow cytometric analysis of intracellular glutathione content (presented as ΔMFI) of stimulated SF and PB CD4^+^ T cells. *N* = 9, 9 independently performed experiments. (D) Statistical analysis of MFI of ROS in stimulated SF and PB CD4^+^ T cells (*N* = 4), 4 independently performed experiments. The calculated power of this experiment is 0.85. (E) Lipid peroxidation as assessed by flow cytometric measurement of anti-CD3/CD28 stimulated CD4^+^ T cells derived from PBMCs and SFMCs. *N* = 11. (F) Gene set enrichment analysis (GSEA) was performed on differentially expressed genes from SF-derived CD4^+^ T cells of patients with active JIA compared to PBMC-derived CD4^+^ T cells from healthy controls. The enrichment plots shown represent driver ferroptosis-related gene sets from KEGG, WikiPathways, and FerrDb V2. (*N* = 4). (G) Heat maps showing normalized counts of selected RNAs determined by RNA-seq in JIA CD4^+^ T cells compared to HC CD4^+^ T cells. *N* = 4 in each group. (H) SFMCs and PBMCs were analyzed for *ASCT1* expression by quantitative RT-PCR. The calculated power of this experiment is 0.99. (I) Flow cytometric analysis of CD36 expression of stimulated SF and PB CD4^+^ T cells. *N* = 5, 5 independently performed experiments. (J) Representative histograms showing BODIPY Ferroptosis staining of anti-CD3/CD28 stimulated HC PBMCs in the absence and presence of ASCT1/2 inhibition by 4-Hydroxy-L-phenylglycin (HPG) and SAS. *N* = 3, 3 independent experiments. The statistical evaluation of (A), (C), (E), (H), and (I) from the represented graphs was performed using a paired Student’s t-test. One-way ANOVA multiple comparison was used for (B) and (D) of the represented graphs. Data are presented as mean, error bars present ± SEM for all the presented graphs in this figure. Values were considered significant if ∗*p* < 0.05, ∗∗*p* < 0.01, ∗∗∗*p* < 0.001, and ∗∗∗∗*p* < 0.0001. N represents the number of biological replicates.

The observation that T cells survive despite SLC7A11 inhibition and produce lower amounts of inflammatory cytokines raises questions about regulations in inflammatory settings. To investigate this, we analyzed T cells from the inflamed joints of children with juvenile idiopathic arthritis. We have previously shown that these SF T cells express diminished levels of NRF2 protein, high levels of ROS, and pro-inflammatory cytokines such as IFN-γ and IL-17.^12^^,^^13^^,^^15^ Consistent with reduced NRF2 levels, SLC7A11 expression of anti-CD3/CD28 stimulated SF CD4^+^ T cells was also reduced (Figure 4A). SFMCs had a tendentially reduced cystine uptake, and inhibition of SLC7A11 with either SAS or erastin did not significantly further downregulate cystine uptake of SFMCs (Figure 4B compared to Figure 2A), which might be explained by low expression of SLC7A11. GSH levels were lower compared to PBMCs, which may indicate enhanced consumption due to increased levels of ROS (Figures 4C and 4D). As for SFMCs, the measurement with BODIPY 581/591 C11 revealed a high autofluorescence in the PE channel (Figures S1B and S1C). We used another dye to check for lipid peroxidation. By doing so, SF T cells showed increased lipid peroxidation compared to PB T cells (Figure 4E) and also revealed a distinct gene expression profile, including Ferroptosis driver pathways, compared to healthy PB T cells (Figure 4F). Stimulated JIA SFMCs downregulate several genes related to ferroptosis compared to stimulated PBMCs. Genes associated with antioxidant processes, such as *GPX4, GSS, GCLC, and SAT2*, are downregulated. These genes are central to GSH biosynthesis and activity.^5^ Genes responsible for iron management, such as *FTL, PCBP1, PCBP2, SLC40A1 (ferroportin)*, and *SLC39A14,* are downregulated. This promotes the accumulation of free labile iron inside the cell, thereby actively increasing ROS levels and lipid peroxidation.^16^^,^^17^ Iron recycling is impaired by the downregulation of *ATG5*, while mitochondrial function is impaired by *VDAC2, VDAC3,* and *ASCL6*, which are important for mitochondrial function and FA handling. Conversely, they upregulate genes such as *MAP1LC3B* and *ATG7*, which are key mediators of ferritin degradation, as well as genes such as *TP53*, *SLC39A8,* and *CP*, which are relevant for increased iron uptake and altered distribution. This results in higher amounts of free iron, which leads to the generation of ROS, the promotion of the Fenton reaction, and the fueling of lipid peroxidation.^2^^,^^17^ The upregulation of the ACSL family, such as *ACSL1, ACSL3, ACSL4*, and ACSL5, together with LPCAT3, increases the generation and incorporation of polyunsaturated fatty acids (PUFAs) into cellular membranes, making them more susceptible to peroxidation.^6^ In summary, the cells appear to actively increase their ROS levels, thereby promoting lipid peroxidation, while attempting to prevent ferroptotic cell death. This is consistent with the upregulation of *GCLM*, which is central to GSH synthesis and limits ROS, thereby buffering the cells against ferroptotic stimuli.^18^ As *GCLC* is downregulated in these cells, *GCLM* is upregulated to increase the enzyme’s affinity for substrates, relieving the feedback inhibition by GSH and potentially compensating for the reduced levels of *GCLC* to balance ROS levels further^19^ (Figure S3A). Considering that despite enrichment of ferroptosis driver genes, higher ROS and lower SLC7A11 expression, SF T cells do not undergo excessive cell death but are even highly inflammatory, we wondered if SF T cells are equipped with compensatory mechanism to prevent ferroptosis. We checked RNA-sequencing data from HC CD4^+^ T cells compared to SF T cells and found a higher expression of *gamma-glutamyltransferase 1 (GGT1)*, *ASCT1* and *ASCT2* (Figure 4G). Levring et al. showed that both SLC7A11 and ASCT1/ASCT2 can provide T cells with the required amounts of Cys^9^ while the so far from us used cystine FITC probe is SLC7A11 and cystine specific.^20^ Interestingly, ASCT1 mRNA was enhanced in SF T cells samples compared with PB T cells (Figure 4H), suggesting a compensatory effect to maintain cellular Cys level. Enhanced GGT1, which is a plasma membrane-bound enzyme that increases Cys availability for cells, might further support SF CD4^+^ T cells to fill their Cys need. CD36, which causes ferroptosis in T cells by mediating oxLDL uptake,^1^^,^^21^ was reduced in SFMCs, which might be the next mechanism to prevent ferroptotic cell death (Figure 4I). Our data thus suggest a delicate balance in SF T cells. SF T cells are characterized by high ROS and low NRF2 with consecutive SLC7A11 levels and higher lipid peroxidation. Nevertheless, SF T cells reveal compensatory characteristics such as low CD36 expression and high *ASCT1* and *GGT1* expression, which most likely prevent ferroptotic cell death. By that, SF T cells survive in an inflammatory milieu with a higher cytokine production instead of undergoing programmed cell death. To finally proof that T cells can switch between either cystine uptake by SLC7A11 or Cys uptake by ASCT1, we inhibited activated HC T cells either with SAS or with the ASCT1 inhibitor HPG, and subsequently measured lipid peroxidation (Figure 4J). Lipid peroxidation increases slightly with each inhibitor and more in combination of both underlying the assumption that HC CD4^+^ T cells can switch between both for their Cys requirements.

## Discussion

Our data suggest that T cells can use an alternative transporter to ensure intracellular Cys levels, as shown before by Levring et al. 9. We also showed that either external inhibition or internal downregulation of the NRF2/SLC7A11 pathway does not induce high levels of ferroptosis in T cells. SF T cells may have a distinct NRF2/SLC7A11 ferroptosis pathway. SLC7A11 expression is downregulated, most likely by reduced NRF2 expression. Reduced NRF2 expression was shown by us before^12^ and was an unexpected observation. A recent study showed that Keap1-mediated NRF2 suppression promotes the adaptation of CD8^+^ T cells to chronic antigen stimulation.^22^ So, the low NRF2 might also be a mechanism of T cells to adapt to chronic inflammation. Despite low NRF2, high intracellular ROS, and an increased demand for antioxidants, lipid peroxidation is only slightly enhanced compared to PB T cells, and these T cells do not undergo programmed cell death but instead produce inflammatory cytokines. High ASCT1 expression in combination with higher GGT1 expression, which facilitates Cys uptake in JIA T cells, could be used as an alternative to cystine uptake by SLC7A11.

The redox regulation of T cells is very complex. Brenner et al. have already shown that the levels of ROS need to be in a certain range, too high levels of ROS are lethal for T cells, while some ROS are required for their proper function. In detail, GSH tempers ROS activity to a sweet spot that allows T cells to enter the cell cycle, reprogram metabolically, differentiate, and form protective immune responses.^8^ In this way, ROS manipulates metabolic processes; too much is disastrous, but the right amount can be lifesaving. In addition, moderate and high overexpression of SLC7A11 exert opposite effects on H_2_O_2_-induced cell death. A disadvantage of SLC7A11 overexpression is glucose and PPP dependence as known from cancer cells.^23^ Moreover, GSH, whose synthesis is mediated via activation of NRF2-regulated genes, itself represses NRF2 activity and might therefore provide a critical self-regulatory feedback mechanism.^24^ Upon activation, T cells usually induce SLC7A11 expression. High SLC7A11 activity requires large amounts of NADPH to avoid cystine accumulation in the cytosol. NADPH is mainly produced in the pentose phosphate pathway. Activated T cells depend on glucose, and glucose levels are reduced in the joint. Lower SLC7A11 expression may therefore be beneficial for T cells to survive in this environment, as a small increase in ferroptosis is accepted to avoid PPP dependence. This may be important as it has recently been shown that JIA SF CD4^+^ T cells exhibit increased glycolytic activity and acetyl-CoA production compared to peripheral blood T cells from the same individual or healthy controls,^25^ in contrast to the metabolic behavior of PB CD4^+^ T cells in patients with RA, which undergo a fundamental shift in glucose utilization, favoring diversion away from pyruvate and lactate production and toward the pentose phosphate pathway.^26^^,^^27^

A second disadvantage of high SLC7A11 expression is SLC7A11-induced glutamine dependence, which results mainly from glutamate-derived anaplerosis.^28^ Besides glucose, glutamine is the most important energy source for T cells; it can either be converted to alpha-KG and used in the TCA, or it can be used for GSH synthesis and transported out of the cell as glutamate. In contrast to SLC7A11, ASCT1 and Cys uptake are not dependent on glutamate, but more on a reducing environment.

Consequently, these two “costs” associated with SLC7A11-mediated cystine uptake, namely glutamate export and high NADPH consumption for cystine reduction, are under tight control in SF T cells. Interestingly, we found that reduction of the NRF2/SLC7A11 pathway and enhanced lipid peroxidation were associated with high levels of inflammatory cytokines, i.e., IL-17 and IFN-γ. The mechanism behind this will be the subject of future studies, as well as a more specific analysis of SAS and erastin exposure on different T cell subsets. Another adapted mechanism to counteract increased lipid peroxidation is reduced CD36 expression in JIA T cells. FA facilitation would actively lead to lipid peroxidation, which is a hallmark of ferroptosis, at least as shown for CD8 T cells in cancer.^1^

We hypothesize that JIA T cells might compensate for the low SLC7A11 expression by upregulation of ASCT1. So far, we only know that Cystine is the dominant form in plasma (90%); it is not clear if the concentrations of cystine and Cys differ from plasma in SF. SFMC could compensate for cystine uptake by upregulating ASCT1, which takes up Cys instead. Cys can be directly converted to GSH in order to tightly control lipid peroxidation.

Nevertheless, T cells are eager to counterbalance redox metabolism in order to prevent ferroptosis, a process involving the ASCT1 and SLC7A11 receptors, as well as the subsequent uptake of Cys and cystine. The expression of these receptors increases once they are activated, which in turn increases the uptake of these amino acids. Without activation, their Cys transport is limited, which is why, in early immune responses, antigen-presenting cells (APCs) import cystine and export Cys so that T cells can take it up. Subsequently, T cells become more autonomous by inducing their own uptake systems, which highlights the important role of ASCT1 in sustaining T cell proliferation and cytokine production.^9^ Cys that is imported into the cell can directly buffer ROS, but it is also important for the functional sites of proteins due to the free thiol group contained within highly conserved residues.^29^ In inflammatory conditions, T cells compete with other cell types for Cys, as they require high amounts, which are typically released by APCs, such as DCs. However, in persistent inflammatory conditions such as cancer or chronic conditions, other cells, such as cancer cells or MDSCs, are actively incorporating that Cys, thereby limiting the extracellular pool of Cys. This may explain why ASCT1 is upregulated to import as much Cys as possible.^30^

So far, SAS is usually used in HLA-B27 positive enthesitis-associated childhood arthritis, but it is also sometimes part of a multi-target DMARD therapy together with methotrexate (MTX) in other forms of childhood arthritis. The low expression of SLC7A11 in SF T cells might explain the therapeutic failure in oligoarticular JIA. Our data reveal that a common inhibition of SLC7A11 and ASCT1 is necessary to efficiently downregulate T cell activation. A recent study from Zhou et al. revealed a role for SLC7A11 as an H+ transporter. SLC7A11 deficiency or inhibition caused lysosomal over-acidification. This function of SLC7A11 in T cells in inflammation will be an interesting point to analyze in the future.^31^

In conclusion, we have analyzed the highly complex network of NRF2/SLCA11-regulated ferroptosis in T cells. Recently, several pharmacological or natural compounds and cell-intrinsic proteins have been reported to regulate the process and function of ferroptotic cell death, and most classical ferroptosis activators are inhibitors of the antioxidant system. Therefore, it is important to understand the network of antioxidant proteins that inhibit ferroptotic cell death.^2^

### Limitations of the study

The constraints of this study are evident in the fact that we were only able to measure a limited cohort of patients. Patients must agree to the use of their material in research, and the amount of fluid that can be extracted from the joint varies considerably between the patients, with some patients unable to provide any fluid. Further studies need to clarify gender-specific differences, which was due to a small number of patients not possible in our study. Most of the patients are female, as the predominance of autoimmune diseases is higher in that cohort, so we could not compare sex influences in this study. We appreciate that western blots of the targets would have been valuable, but it is not possible to include them at this time due to the amount of material we received. However, this will be a focus in the future. In addition, there is no *in vivo* model that reflects juvenile arthritis and could further support our hypothesis. Although we identified ASCT1 as a key regulator in JIA T cells, we lack the ability to fully uncover the interplay between SLC7A11/ASCT1 and the cystine/Cys uptake switch. Even though we provide a full explanation that the inhibitors increase ferroptosis and diminish cystine uptake, we cannot fully explain the decrease in ROS levels in contact with at least erastin. While the inhibitors appear to increase ROS in Jurkat cells, they appear to decrease ROS levels in humans while having no effects in mice (data not shown). This suggests that cells can activate another layer of compensation, which will be the focus of future studies. We tried to introduce a knock-down via siRNA and neon transfection, which we could achieve with HC PBMCs by 60% but not SFMCs. We believe this is due to high apoptosis rates during electroporation. In future studies, we want to test knockdowns with lentiviral transductions.

## Resource availability

### Lead contact

Request for further information and resources should be directed to and will be fulfilled by the lead contact, Kim Ohl (kim.ohl@rwth-aachen.de).

### Materials availability

This study did not generate new unique reagents.

### Data and code availability

• RNAseq data were uploaded to the GEO database: GSE161426 and was already published in ref.^32^
• The microarray data were uploaded to the GEO database: GSE325439.
• The paper does not report original code.
• Data is available upon reasonable request to the lead contact. All the relevant data generated during this study is included in the article.

## Acknowledgments

This work was supported by the Flow Cytometry and the Genomics Facility, both being a core facility of the Interdisciplinary Center for Clinical Research (IZKF) Aachen within the Faculty of Medicine at RWTH Aachen University. The study was funded by ERA-PerMed 2020-305 PerMIDRIAR to K.T.

The graphical abstract was created in BioRender. Ohl, K. (2026) https://BioRender.com/w7ocm5m.

## Author contributions

C.T.N. drafted and critically revised the article. He measured, analyzed, and interpreted data except for some of the data mentioned later in discussion. S.H.S. contributed significantly to the acquisition and analysis of 4-OI and siRNA data. G.H. provided substantial contributions to the acquisition of data. T.K. substantially contributed to the acquisition of data. F.H. substantially contributed to the analysis of data, J.v.L. substantially contributed to the acquisition of data. B.V. substantially contributed to the analysis and interpretation. K.T. substantially contributed to the analysis and interpretation of data. K.O. substantially contributed to the study conception and design, and data analysis. All authors contributed critically revising the article for important intellectual content and finally approved the version of the article to be published.

## Declaration of interests

The authors declare no competing interests.

## STAR★Methods

### Key resources table

**Table:** 

REAGENT or RESOURCE	SOURCE	IDENTIFIER
**Antibodies**		

Cell Proliferation Dye eFluor670	ThermoFisher Scientific	65-0840-85
anti-CD3	ThermoFisher Scientific	16-0037-85 (RRID:AB_468855)
anti-CD28	ThermoFisher Scientific	16-0289-85 (RRID:AB_468927)
eBioscience™ Fixable Viability Dye eFluor™ 520	Invitrogen	65-0867-14
anti-XCt	Abcam	ab216876 (RRID:AB_3678921)
goat anti-rabbit IgG FITC	BD Pharmingen	554020 (RRID:AB_395212)
Ki-67 FITC	Invitrogen	11-5698-82 (RRID:AB_11151330)
INFg APC	Invitrogen	17-7319-82 (RRID:AB_469506)
IL-17 PE	Invitrogen	12-7179-42 (RRID:AB_1724136)
IL-4 Alexa Fluor^TM^ 488	Invitrogen	53-7049-42 (RRID:AB_1724142)
HELIOS eFluor^TM^ 450	Invitrogen	48-9883-42 (RRID:AB_2574136)
FoxP3 APC	Invitrogen	17-4776-42 (RRID:AB_1603280)
CD4 eFluor^TM^ 450	Invitrogen	48-0048-42 (RRID:AB_2016674)
CD4 PE	Invitrogen	12-0048-42 (RRID:AB_2016675)

**Chemicals, peptides, and recombinant proteins**		

Ficoll gradient	PAN Biotech	P04-601000
RPMI 1640 medium	ThermoFisher Scientific	21875-034
10% heat-inactivated FBS	ThermoFisher Scientific	10500-064
1% penicillin/streptomycin	ThermoFisher Scientific	15140-122
sulfasalazine	Sigma Aldrich	S0883
erastin	Sigma Aldrich	E7781
PMA	Sigma Aldrich	P1585
ionomycin	Sigma Aldrich	I0634
GolgiStop^TM^	BD Bioscience	555029
GolgiPlug^TM^	BD Bioscience	554724
RPMI 1640 ATCC medium	ThermoFisher Scientific	A1049101
NRF2 siRNA	OriGene	SR321100
RNeasy Mini Kit	Qiagen	74104
First Strand cDNA Synthesis Kit	Thermo Fisher Scientific	K1621
SYBR Green	BioRad	1725271
Neon transfection	ThermoFisher Scientific	MPK10025

**Critical commercial assays**		

EBioscience™ Annexin V Apoptosis Detection Kits	Invitrogen	88-8103-74 (RRID:AB_2575186)
FOXP3/Transcription factor staining buffer set	ThermoFisher Scientific	00-5523-00
Biotracker^TM^ Cystine-FITC Live Cell Dye	Merck	SCT047
GSH detection kit	Abcam	ab11232
ROS Assay Kit 520 nm	Invitrogen	88-5930-74 (RRID:AB_2574932)
MitoTracker™ Green FM	Invitrogen	M7514
tetramethylrhodamine methyl ester (TMRM) Detection kit	Invitrogen	M20036
BODIPY 581/591 C11	Invitrogen	D3861
Click-iT Lipid Peroxidation Detection Kit	Invitrogen	C10446
GSH/GSSG-Glo™ Assay	Promega	V6611

**Deposited data**		

RNA-seq data already published	Conserved human effector Treg cell transcriptomic and epigenetic signature in arthritic joint inflammation - PMC	GEO database: GSE161426

**Experimental models: Cell lines**		

Jurkat cells	N/A	RRID:CVCL_0065

**Experimental models: Organisms/strains**		

C57BL/6 Keap^fl/fl^	Okawa et al.^33^	N/A
C57BL/6 *VAV*^*cre*^*Keap*^*fl/f*^	Kim et al.^34^	N/A
NRF2-KO	Chan et al.^35^	N/A

**Oligonucleotides**		

*SLC7A11*	F: 5′ TGA CTG GAGTCC CTG CGT AT 3′; R: 5′ TGT TCT GGT TAT TTT CTC CGA CA 3′	eurofins
*b-Actin*	F: 5′ AGA TGG CCA CGG CTG CT 3′; R: 5′ AAC CGC TCA TTG CCA ATG G 3′	eurofins
*ASCT1*	F: 5′-ATT GGT CCT GTT TGC TCT GG-3′; R: 5′-TGG GGA GAA TAA ACC TGC TG-3′	eurofins
*ASCT2*	F: 5′-TAC ATT CTG TGC TGC CTG CT-3′; R: 5′-ATG AAA CGG CTG ATG TGC TT-3′	eurofins
*LAT1*	F: 5′-GTG ACG CTG GTG TAC GTG CT-3′; R: 5′-GGG TGG ATC ATG GAG AGG AT-3′	eurofins

**Software and algorithms**		

Prism	GraphPad Software	10.6.1
G∗Power	HHU Düsseldorf	3.1.9.7
FlowJo	BD Bioscience	v10.10.0

### Experimental model and study participant details

No cell lines were involved in this study.

### Method details

#### Ethics statement and patients

All patients enrolled were diagnosed with JIA and were receiving nonsteroidal anti-inflammatory drugs prior to therapeutic aspiration of SF and administration of corticosteroids. The use of this material was approved by the local ethics committee of the RWTH Aachen University Hospital (ethical approval reference: 384-19) and written consent was obtained the human subject or their legal guardians. The obtained samples would have been discarded if not used for research purposes. SF was obtained in an anonymized form for research purposes. Cells were pelleted by centrifugation, and the supernatants were stored at -80°C. Blood was drawn along from patients where written consent was obtained by the patient or their legal guardians. Information about sex, gender and age can be found in Table S1. Other information about the patient’s ancestry, ethnicity was not allowed to be collected and stored. The patients are predominantly female, as autoimmune diseases are more prevalent in women. The primary cells were not tested for mycoplasma.

#### Mice

Experiments were performed with sex and age-matched 6 – 10 weeks old Keap^fl/fl^, *VAV*^*cre*^*Keap*^*fl/f*^ mice (all C57BL/6)^34^ and Nrf2-Knock-out mice, which were obtained by specific deletion of the Nrf2 gene segment.^36^ Single cell suspensions were isolated from spleens, and erythrocytes were lysed with lysis buffer. All animal experiments were performed in compliance with the German animal protection law (TierSchG) and approved by the local animal welfare committees and the Landesamt für Verbraucherschutz und Ernährung (LAVE), North Rhine Westfalia (internal numbers: 30277A4, 50199A4). The mice breeding was done in our animal facility. Mice were housed in groups with food and water available *ad libitum.* The animal facility caretaker strictly maintained the light/dark schedule, following a 12:12 scheme. The mice were fed standard control chow until they were 6 to 10 weeks old. The Mice were then sacrificed for cell isolation and further characterisation of the spleenocytes. The primary cells were not tested for mycoplasma.

#### Cell isolation

Human peripheral blood mononuclear cells (PBMCs) from JIA patients and healthy donors were isolated on a Ficoll gradient (PAN Biotech, Aidenbach, Germany; P04-601000). SF was obtained by centrifugation at 400×*g* for 10 min. The pelleted cells were used to isolate synovial fluid mononuclear cells (SFMCs) on a Ficoll gradient.

#### Cell culture

PBMCs from healthy donors were cultured in RPMI 1640 medium (ThermoFisher Scientific (Gibco), Karlsruhe, Germany; 21875-034) supplemented with 10% heat-inactivated FBS (ThermoFisher Scientific (Gibco), Germany; 10500-064) and 1% penicillin/streptomycin (ThermoFisher Scientific (Gibco), Germany; 15140-122). For proliferation assays, PBMCs were labelled with the cell proliferation dye eFluor670 (5 μM) (ThermoFisher Scientific, Germany; 65-0840-85) according to the manufacturer’s instructions. PBMCs were stimulated with 5 μg/mL anti-CD3 (OKT3) and 2 μg/mL anti-CD28 (CD28.2) (ThermoFisher Scientific, Germany; 16-0037-85 and 16-0289-85, RRID:AB_468855 and RRID:AB_468927) respectively, and the cells were treated with 200 μM sulfasalazine (SAS, Sigma Aldrich, Germany, S0883), 10 μM erastin (Sigma Aldrich, Germany, E7781), or DMSO as a vehicle control for 48 hours, unless otherwise indicated.

#### Flow cytometry and antibody staining

PBMCs were stained extracellularly with CD4 eFluor^TM^ 450 (Invitrogen, Germany; clone OKT4, 48-0048-42, RRID:AB_2016674), CD4 PE (Invitrogen, Germany; clone OKT4, 12-0048-42, RRID:AB_2016675). Viability staining was performed using eBioscience^TM^ Fixable Viability Dye eFluor^TM^ 520 (Invitrogen, Germany; 65-0867-14) and EBioscience^TM^ Annexin V Apoptosis Detection Kits (Invitrogen, Germany; 88-8103-74, RRID:AB_2575186) according to the manufacturers protocol. For the analysis of intracellular markers, cells were fixed and permeabilized with a FOXP3/Transcription factor staining buffer set (ThermoFisher Scientific, Germany; 00-5523-00) according to the manufacturer’s instructions. For cytokine staining, stimulated PBMCs were restimulated for 5 hours with PMA (Simga-Aldrich, Germany, P1585) and ionomycin (Sigma-Aldrich, Germany, I0634) including GolgiStop^TM^ (IL-4, BD Bioscience, 555029) or GolgiPlug^TM^ (IFN-γ and IL-17, BD Bioscience, 554724) in cytokine medium containing 1% penicillin/streptomycin RPMI 1640 ATCC medium (ThermoFisher Scientific (Gibco), Karlsruhe, Germany; A1049101). The cells were then stained with anti-XCt (1:1000, Abcam, UK; rabbit polyclonal, ab216876, RRID:AB_3678921), goat anti-rabbit IgG FITC (1:200, BD Pharmingen, Germany; clone RUO, 554020, RRID:AB_395212), Ki-67 FITC (Invitrogen, Germany; clone SolA15, 11-5698-82, RRID:AB_11151330), INFγ APC (Invitrogen, Germany; clone 4S.B3, 17-7319-82, RRID:AB_469506), IL-17 PE (Invitrogen, Germany; clone eBio64DEC17, 12-7179-42, RRID:AB_1724136), IL-4 Alexa Fluor^TM^ 488 (Invitrogen, Germany; clone 8D4-8, 53-7049-42, RRID:AB_1724142), HELIOS eFluor^TM^ 450 (Invitrogen, Germany; clone 22F6, 48-9883-42, RRID:AB_2574136) and FoxP3 APC (Invitrogen, Germany; clone PCH101, 17-4776-42, RRID:AB_1603280).The Biotracker^TM^ Cystine-FITC Live Cell Dye (Merck, Germany; SCT047) was used according to manufactures instructions. Intracellular GSH Detection was performed by harvesting stimulated cells and staining with the intracellular GSH detection kit (1:200, Abcam, UK; ab11232) in RPMI medium containing lineage antibodies. DMSO was used as the FMO control. Samples were incubated at 37°C for 30 minutes, washed, and processed for flow cytometry. Total ROS of stimulated cells was measured using the Total ROS Assay Kit 520 nm (Invitrogen, Germany; 88-5930-74, RRID:AB_2574932) following manufactures instructions. MitoTracker^TM^ Green FM (1:50000, Invitrogen, Germany; M7514) was used to label mitochondria, while the tetramethylrhodamine methyl ester (TMRM) Detection kit (1:2000, Invitrogen, Germany; M20036) was used to label active mitochondria. Ferroptosis was measured with BODIPY 581/591 C11 (1:500, Invitrogen, Germany; D3861). Lipid peroxidation was assessed by the Click-iT Lipid Peroxidation Detection Kit (Invitrogen, Germany; C10446). Cells were stimulated with Click-iT LAA solution (1:1000) as described above. Cells were harvested and stained according to the manufacturers protocol.

Flow cytometry was performed with the FACSCanto II or LSRFortessa instrument (BD Biosciences, Germany). Data analysis was performed using FlowJo^TM^ software (for Windows) version *10.10.0* (Becton, Dickinson and Company; 2023). For the gating strategies applied to the data, please refer to Figures S1 and S2, as indicated in the figure captions.

#### siRNA experiments

Briefly, 2.5 × 10^6^ cells were suspended in T cell suspension buffer. To this, 10 nM NRF2 siRNA (OriGene, Germany; catalogue number SR321100) or a control was added, and transfection was performed according to the manufacturer’s instructions (Neon transfection, ThermoFisher Scientific; MPK10025). After transfection, the transfected cells were added with RPMI + 10% FCS (without antibiotics) in a 12-well culture plate and incubated at 37°C for 24 hours. After 24 h incubation, the transfected cells were stimulated with anti-CD3 (5 μg/mL) and anti-CD28 (1 μg/mL) antibodies for 24 h in a 96-well U-bottom plate. After the final incubation, the cells were used for further analysis.

#### RT-qPCR

Total RNA was extracted from cells using a RNeasy Mini Kit (Qiagen, Germany, 74104) and transcribed into cDNA using a First Strand cDNA Synthesis Kit (Thermo Fisher Scientific, USA, K1621) according to the manufacturer’s instructions. Standard quantitative real-time PCR (RT-qPCR) was performed on a TaqMan 7900 (Applied Biosystems, USA) using the DNA intercalating dye SYBR Green (BioRad, 1725271). Each measurement was performed in duplicate and relative expression was calculated after normalization to the endogenous housekeeping control gene *β-Actin.* The following primers were used: forward (F): 5′ TGA CTG GAGTCC CTG CGT AT 3′, reversed (R): 5′ TGT TCT GGT TAT TTT CTC CGA CA 3′; *SLC7A11*, F: 5′ AGA TGG CCA CGG CTG CT 3′, R: 5′ AAC CGC TCA TTG CCA ATG G 3′; *β-Actin*, F: 5′-ATT GGT CCT GTT TGC TCT GG-3′, R: 5′-TGG GGA GAA TAA ACC TGC TG-3′; *ASCT1*, F: 5′-TAC ATT CTG TGC TGC CTG CT-3′, R: 5′-ATG AAA CGG CTG ATG TGC TT-3′; *ASCT2* and F: 5′-GTG ACG CTG GTG TAC GTG CT-3′, R: 5′-GGG TGG ATC ATG GAG AGG AT-3′; *LAT1*.

#### RNA extraction and microarray for gene expression analysis

Genome-wide transcriptome analyses for *VAV*^*cre*^*Keap*^*fl/fl*^ (Keap1-KO) and *VAV*^*cre*−^*Keap*^*fl/fl*^ (WT)CD4+ T cells were performed in independent quadrigatus using Gene Chip® Mouse Gene 2.0 arrays. Data was analysed by a bioinformatician during the sequencing service request by the Genomics Facility of the Interdisciplinary Center for Clinical Research (IZKF) within the Faculty of Medicine at RWTH Aachen University.

#### GSEA ferroptosis pathway

RNA-sequencing data used for gene set enrichment analysis are publicly available under accession number GSE161426 in the NCBI Gene Expression Omnibus (GEO). The analysis focused on the *adultPBTef* and *JIASFtef* sample groups, representing effector CD4^+^ T cells isolated from healthy adult peripheral blood and from the synovial fluid of patients with juvenile idiopathic arthritis (JIA), respectively. Detailed information on sample collection, processing, and sequencing protocols is available in the original publication.^32^

Gene set enrichment analysis was performed using the fgsea R package (v1.32.0). Genes were ranked by log_2_ fold change. To resolve tied ranks, small random noise was introduced, preserving the overall ranking structure. Gene sets were sourced from the KEGG, WikiPathways and FerrDb V2 (human) collections, covering both ferroptosis-associated driver and suppressor genes (Table S2). As the functional role of some genes is context dependent, individual genes may appear in both driver and suppressor sets, following FerrDb V2 annotation. The fgsea() function was applied using the complete KEGG and WikiPathways collections as background to ensure accurate calculation of adjusted p-values for all enrichment testing. Normalized enrichment scores (NES) and statistical significance were computed. Enrichment plots for ferroptosis pathways were generated using the plotEnrichment() function.

#### GSH/GSSG-Glo^TM^ assay

Freshly isolated HC PBMCs were positively selected for CD4 via CD4 Microbeads (130-045-101, Miltenyi Biotech), according to the manufacturer’s instructions. 50,000 cells per well were incubated with either control, SAS, or erastin, as previously described. After two days of stimulation, the cells were immediately measured for GSH and GSSG content using a Luminometer. The measurement and the calculation of GSH/GSSG ratio was conducted according to the manufacturer’s instructions (V6611, Promega).

### Quantification and statistical analysis

Flow cytometric analysis and gating was performed using FlowJO (BD Bioscience, v10.10).

Results are expressed as the mean ± standard error of the mean (SEM). Differences between groups were assessed using two-tailed paired Student’s t-test if the data were normally distributed or using One-Way ANOVA multiple comparison. Values were considered significant if ∗ p < 0.05, ∗∗ p < 0.01, ∗∗∗ p < 0.001, and ∗∗∗∗ p < 0.0001. All statistical analysis and subsequent graphics generation were performed using GraphPad Prism (GraphPad Software, USA, 10.6.1). Statistical power analysis was performed using G∗Power 3.1.9.7 software.
