# Supplementary material for: Inflammatory CD4+ T cells can waive NRF2-dependent SLC7A11-mediated cystine uptake by using ASCT1
Source: iScience. 2026 Apr 12;29(5):115680. doi: 10.1016/j.isci.2026.115680 (PMC13138027; doi:10.1016/j.isci.2026.115680)
Supplement: Document S1. Figures S1–S3 and Tables S1 and S2 [file mmc1.pdf]

iScience, Volume *29*

Supplemental information

Inflammatory CD4$^+$ T cells can waive NRF2-dependent SLC7A11-mediated cystine uptake by using ASCT1

Christopher Thomas Neullens, Sudheendra Hebbar Subramanyam, Gerd Horneff, Tilmann Kallinich, Freya Huijsmans, Jorg van Loosdregt, Bas Vastert, Klaus Tenbrock, and Kim Ohl

Supplementary Figures

Supplementary Figure 1

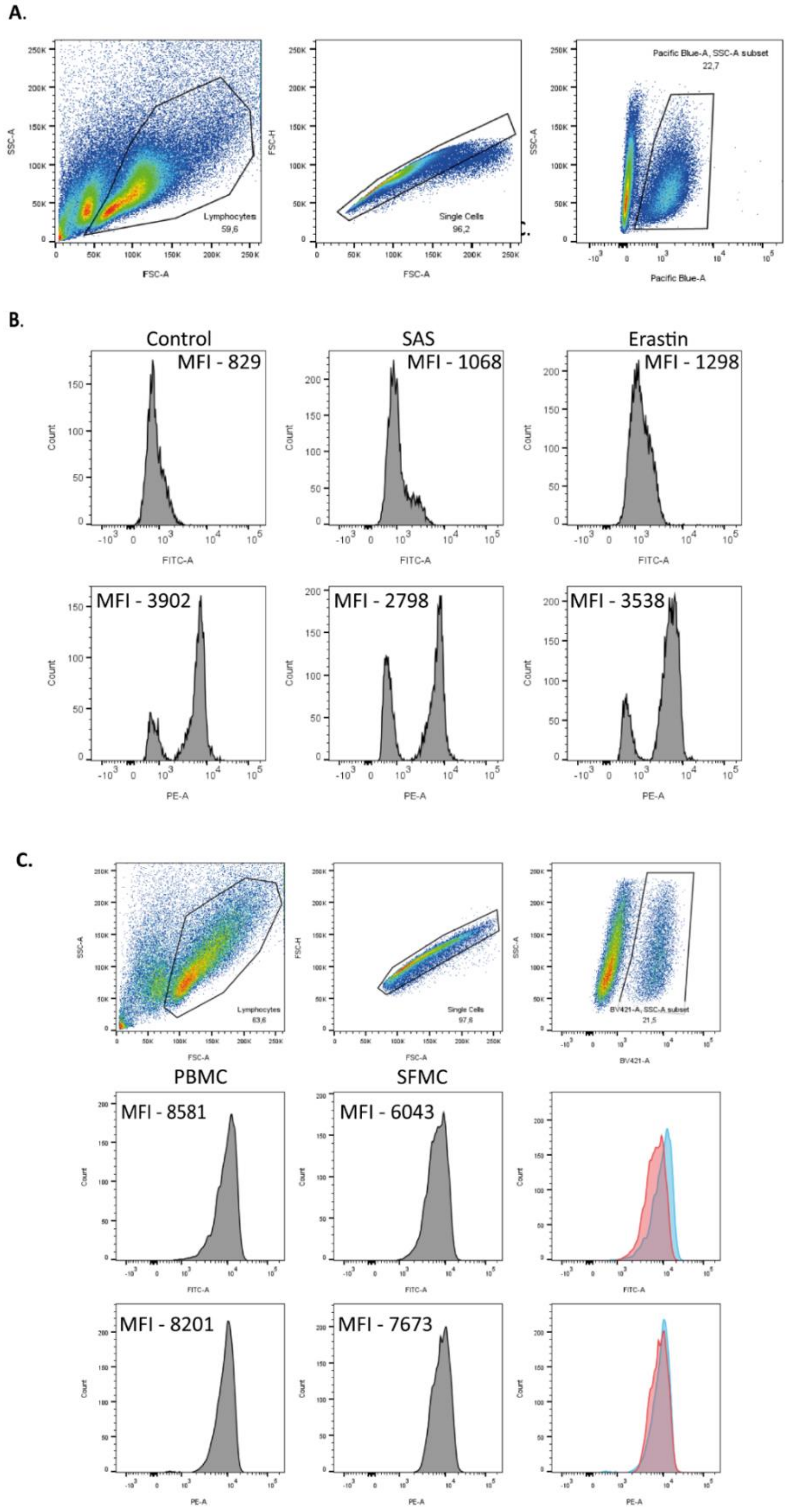

Supplementary Figure 1: The different staining panels are all gated on CD4 T cells, which is shown here as the gating strategy (A). First, we measured ferroptosis using the dye BODIPY C11 581/591. When ferroptosis is increased, the dye is cleaved and its fluorescence is shifted from the PE into the FITC channel, as indicated here by the ferroptosis inducers SAS and erastin (B). When using SFMC, ferroptosis cannot be measured with this dye. The PE channel indicates higher ferroptosis in SFMCs, while the signal in the FITC channel also decreases. Therefore, we switched to the lipid peroxidation kit for these samples.

Supplementary Figure 2

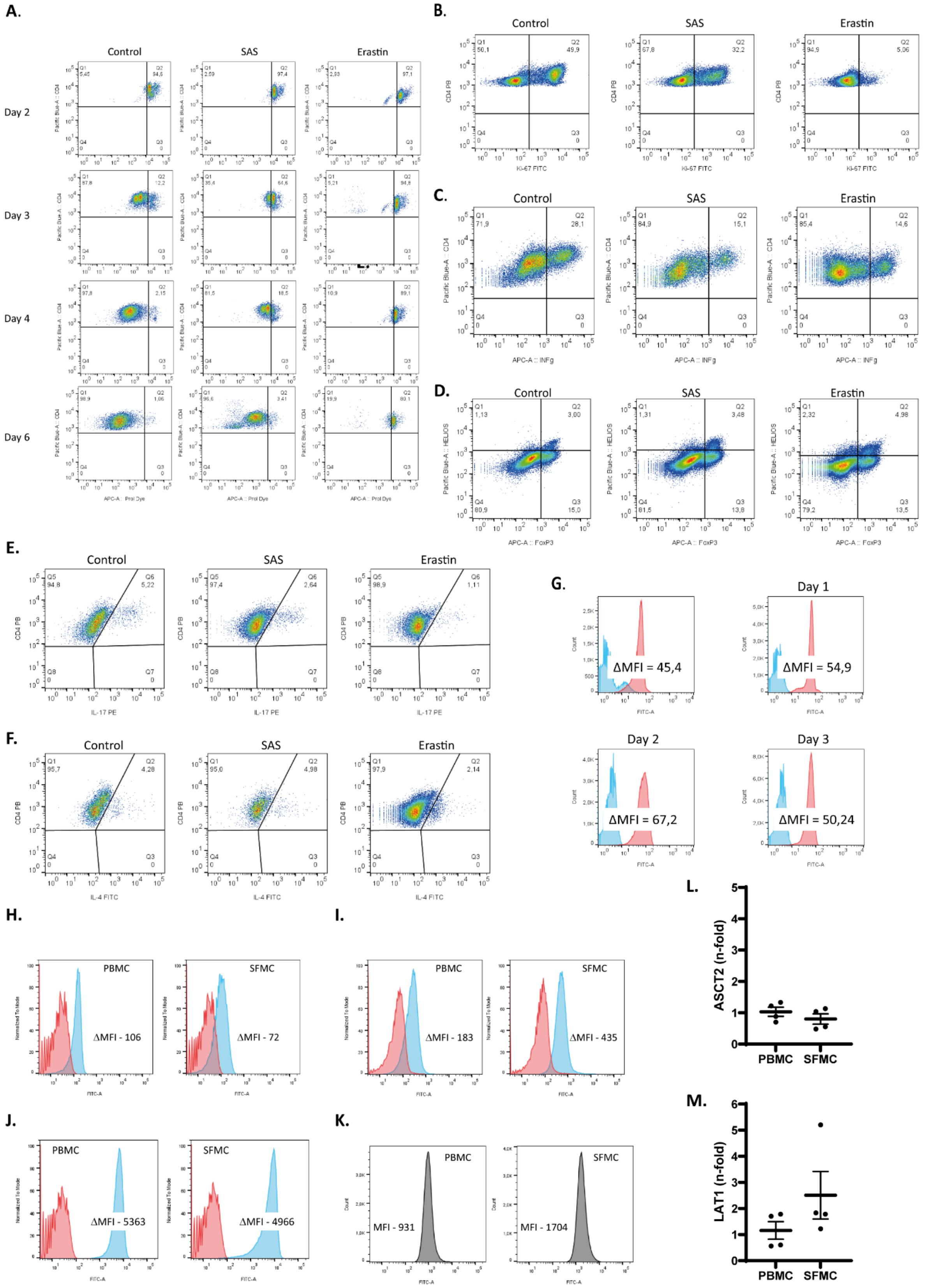

Supplementary Figure 2: The different gating strategies of several dyes used in Figure 3 are shown here: Proliferation dye (A), Ki-67 (B), INFγ (C), HELIOS FoxP3 positive cells (D), IL-17 (E), IL-4 (F), SLC7A11 within different timepoints of stimulation (G), SLC7A11 in SFMC vs PBMC (H), cystine uptake overlay of 0 minutes and 30 minutes (I), GSH detection (J), and lipid peroxidation (K). Other amino acid transporters are not different to PB T cells such as ASCT2 (L) or LAT1 (M).

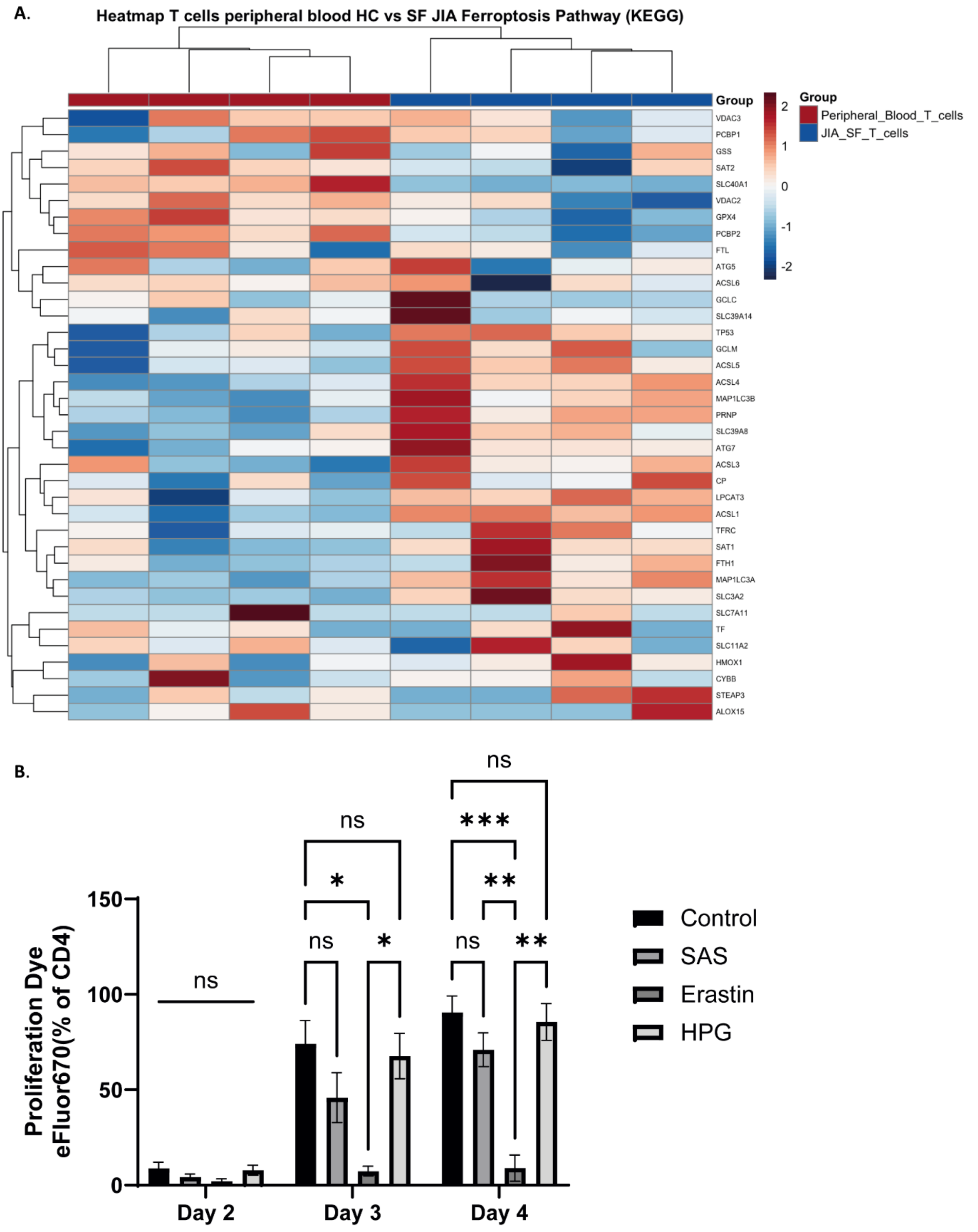

Supplementary Figure 3: Heatmap up- and downregulated genes within the Ferroptosis Pathway by KEGG comparing HC CD4 T cells with SF JIA CD4 T cells (A). Proliferation of a-CD3/CD28 stimulation SFMC with and without inhibition with either SAS, erastin, or HPG (B).

Supplementary Table 1: This is the patient information for the human material used in this study. This includes information on the patient's sex, year of birth, date of receipt and type of sample.

Patient information				
**No.**	**sex**	**Age (y)**	**received**	**material**
1	female	2	Dez 23	PBMC + SFMC
2	female*	6	Mai 24	PBMC + SFMC
3	female	8	Jun 24	SFMC
4	female*	6	Jun 24	PBMC + SFMC
5	female	4	Aug 24	PBMC + SFMC
6	female	22	Sep 24	SFMC
7	female	5	Nov 24	PBMC + SFMC
8	female	5	Dez 24	SFMC
9	female	3	Jan 25	PBMC + SFMC
10	male	16	Jan 25	SFMC
11	female	14	Mar 25	PBMC + SFMC
12	female	17	Apr 25	PBMC + SFMC
*** Same donor received twice**				

Supplementary Table 2: GSEA results of ferroptosis pathway in SF-derived CD4^{+} T-cells of active JIA patients compared to PBMC derived CD4^{+} T-cells of HCs.

Ferroptosis pathway	pval	padj	log2err	ES	NES	size
**KEGG_drivers**	0.00194	**0.033642**	0.45506	0.65326	1.87389	24
KEGG_drivers_and_suppressors	0.09845	0.341078	0.18643	0.43461	1.3724	37
KEGG_suppressors	0.66367	0.865183	0.05973	0.30938	0.84887	20
**Wikipathways_drivers**	0.00107	**0.023541**	0.45506	0.62136	1.86244	29
Wikipathways_drivers_and_suppressors	0.26207	0.584086	0.10797	0.33365	1.11497	57
Wikipathways_suppressors	0.76963	0.89947	0.05143	0.25614	0.80198	36
FerrDb_V2_human_drivers	0.0163	0.119559	0.35249	0.33443	1.3666	203
FerrDb_V2_human_drivers_and_suppressors	0.01865	0.128064	0.35249	0.29553	1.28468	434
FerrDb_V2_human_suppressors	0.0977	0.341078	0.16957	0.28883	1.20058	257
**Wikipathways_KEGG_FerrDb_V2_combined_drivers**	0.00351	**0.044999**	0.43171	0.34911	1.42909	209
Wikipathways_KEGG_FerrDb_V2_combined_drivers_and_suppressor	0.00674	0.071324	0.40702	0.3047	1.33189	447
